# Eye blinks are perceived as communicative signals in human face-to-face interaction

**DOI:** 10.1371/journal.pone.0208030

**Published:** 2018-12-12

**Authors:** Paul Hömke, Judith Holler, Stephen C. Levinson

**Affiliations:** 1 Language and Cognition Department, Max Planck Institute for Psycholinguistics, Nijmegen, The Netherlands; 2 Donders Institute for Brain, Cognition and Behaviour, Radboud University Nijmegen, The Netherlands; 3 Centre for Language Studies, Radboud University Nijmegen, The Netherlands; Arizona State University, UNITED STATES

## Abstract

In face-to-face communication, recurring intervals of mutual gaze allow listeners to provide speakers with visual feedback (e.g. nodding). Here, we investigate the potential feedback function of one of the subtlest of human movements—eye blinking. While blinking tends to be subliminal, the significance of mutual gaze in human interaction raises the question whether the interruption of mutual gaze through blinking may also be communicative. To answer this question, we developed a novel, virtual reality-based experimental paradigm, which enabled us to selectively manipulate blinking in a virtual listener, creating small differences in blink duration resulting in ‘short’ (208 ms) and ‘long’ (607 ms) blinks. We found that speakers unconsciously took into account the subtle differences in listeners’ blink duration, producing substantially shorter answers in response to long listener blinks. Our findings suggest that, in addition to physiological, perceptual and cognitive functions, listener blinks are also perceived as communicative signals, directly influencing speakers’ communicative behavior in face-to-face communication. More generally, these findings may be interpreted as shedding new light on the evolutionary origins of mental-state signaling, which is a crucial ingredient for achieving mutual understanding in everyday social interaction.

## Introduction

Human communication is a joint activity [1]. Rather than just one party being active at a time by producing speech, both speaker *and listener* contribute signals critical to progressing the exchange of information. Listener feedback (or ‘back-channel’ responses [2]), such as ‘*mhm’* or ‘*uhu’*, are crucial for successful communication since they facilitate the process of grounding [3] and thus the achievement of mutual understanding. Eliminating or reducing listener feedback is detrimental to speakers’ behavior [4].

Unlike other animals, humans tend to engage in mutual gaze when communicating without necessarily signaling aggressive intent or affiliative interest [5,6]. For successful face-to-face communication, these recurring intervals of mutual gaze are important, as they allow listeners to also provide speakers with *visual* feedback, such as nodding. Here, we investigate a behavior that—unlike nodding—is not commonly known for its communicative function, or for its role in the process of grounding: eye blinking.

Infants hardly blink [7], but blink rate increases over time until adulthood [8]. Adults blink more frequently than physiologically required for ocular lubrication [9]. We blink approximately 13,500 times every day—thus making it the most frequent facial action—with blinks being among the fastest movements the human body can generate [10]. In addition to reflex protective and physiological eye-wetting functions [9], blinking has been shown to index cognitive load. Under low cognitive load, people blink more than under high cognitive load [7,11]. A neuroimaging study has corroborated these behavioral findings showing that blinking deactivates the dorsal attention network while activating the default-mode network, suggesting an active involvement of blinking in attentional disengagement [12]. Blinking has also been linked to social functions. Comparing different species of non-human primates has revealed that blink rate correlates positively with social group size [13], a measure of social complexity associated with neocortex size (evidence used to support the “social brain hypothesis” [14]. Further, comparing different activity types in humans—that is, looking at a still target, reading, and conversation—the highest blink rate was found in conversation [15]. This suggests that in conversation blinking may fulfill functions beyond physiological and cognitive functions.

A recently published study corroborates this suggestion by taking a close look at the statistical distribution of eye blinking in face-to-face conversation [16]. While our intuitive assumption may be that eye blinking is something we do at entirely random points while we listen to someone else speak, the study revealed the opposite to be the case: the majority of listener eye blinks occurred in typical ‘feedback slots’ during conversation, namely around the end of speaking units (or turn-constructional units, i.e., junctures at which a speaking turn may be perceived as possibly complete [17]). Voluntary blinks have longer durations [18] and longer blinks have been suggested to have a special communicative salience [19,20]. Hömke et al. [16] therefore categorized blinks into short and long listener blinks, based on the distribution of blink durations observed in their corpus of face-to-face conversations, using a threshold of 410 ms, separating the longest 25% from the rest. Long listener blinks (see S1 Video for an example) were primarily produced during mutual gaze, in specific communicative contexts, and also in co-occurrence with other listener feedback responses like nods. Together, these findings suggest that even extremely subtle movements such as eye blinks may be perceived as meaningful signals by others. If this is indeed so, then we should be able to pinpoint some direct communicative consequences that result from the perception of listeners’ eye blinks. However, the corpus data observed by Hömke et al. [16] is limited in the extent to which it can provide such evidence due to the correlational nature of corpus studies. Here, we build on this earlier research by experimentally testing whether listener blink behavior has any measurable effect on speakers’ speech production.

Questions regarding the causal role of subtle facial cues in interactive face-to-face communication have previously been impossible to address with a high degree of experimental control. Hence, we developed a novel experimental paradigm using Virtual Reality technology enabling us to selectively manipulate blink duration in a virtual listener. Participants were asked to have a conversation with three different avatars and to respond to open questions (e.g., *How was your weekend*, *what did you do*?). While participants were answering, the avatar produced different types of visual feedback, which was triggered secretly by a confederate, who could see and hear the participant (via a video-camera link) but who was blind to the conditions and hypotheses and instructed to press a button whenever it felt appropriate to signal understanding. The confederate’s button presses triggered either (1) no listener feedback in the avatar (control condition), (2) nods with short blinks or (3) the same nods but with slightly longer blinks. The nods accompanied the avatar’s blinking behavior to mimic the typical natural environment of blinks that occur in feedback slots in conversation [16]. Crucially, the nods in the two conditions were identical in form and duration such that the only difference between the two conditions was the duration of the co-occurring blinks.

The temporal length of participants’ answers was compared across conditions from the first to the last vocalization produced by the speaker in response to each question. The rationale was that if listener blink duration is irrelevant for the speaker’s speaking behavior, one should not observe any differences in answer length between the nod with short blink and the nod with long blink condition. Alternatively, if listener blinking can indeed function communicatively, then differences in speaker behavior would be expected. Specifically, if nods with long blinks indeed function as a “move on” signal of understanding, signaling “I’ve received enough information for current purposes”, as has been suggested by Hömke et al. [16], answers should be shorter in the nod with long blink than in the nod with short blink condition.

Speaking behavior, like any other social behavior, varies from individual to individual [21]. One particular individual difference measure of dispositional social sensitivity—the Empathy Quotient [22]—may modulate the perception of eye blinks. That is, sensitivity to blink feedback may depend on the speaker’s degree of empathy, which is the “drive or ability to attribute mental states to another person/animal, and entails an appropriate affective response in the observer to the other person’s mental state” [22]. It has been observed that “to drive your point home in a discussion for far longer than is sensitive to your listener” constitutes low-empathy behavior [22], suggesting that high-empathy speakers may be more sensitive to listener feedback in general. More specifically, it has been shown that, passively watching another person telling a story on video, brains of high-empathizers were more responsive (i.e., showed a higher amplitude) to the narrator’s spontaneous eye blinks than brains of low-empathizers (the measured brain responses to observed blinks were magnetoencephalographic and they peaked at about 250 ms in the parieto-occipital cortex [23]). Interestingly, the brain response to observed blinks was only modulated by empathy when the video of the person telling the story was presented with sound, “most likely because the human voice created a social context that enforced the empathy-related modulation on brain activity” [23]. These findings suggest that high-empathizers may be especially responsive to (variations of) blink behavior in face-to-face communication. To address this issue, the random sample of healthy participants in our study was asked to complete the Empathy Quotient questionnaire [22] after the experiment.

The overall aim of the current study was to experimentally test earlier claims based on correlational evidence suggesting that listener blinking may serve a communicative function in conversation [16]. Our hypothesis was that speakers would produce shorter answers when talking to a listener providing feedback in the form of nods with long instead of short blinks (and that this effect may be modulated by speakers’ empathy). The present study demonstrates, for the first time, a sensitivity of speakers to listener blink behavior as a communicative signal in interactive face-to-face communication.

## Materials and methods

### Participants

Forty native Dutch speakers were recruited through the MPI for Psycholinguistics—subject database (www.mpi.nl/ppreg) to participate in the experiment. Data of four participants could not be used due to technical error. Data of one additional participant were excluded from all analyses because he excessively looked away from the screen (more often than 2.5 SD above the mean) during avatar listener responses (and therefore could not have been influenced by them), resulting in a final sample of 35 participants (18–38 years; mean age = 21.88; 19 females, 16 males). Our sample size was planned in accordance with prior studies on listener feedback [24]. The whole session lasted about sixty minutes and each participant was paid €10.

### Apparatus and materials

#### Laboratory set-up and equipment

**Fig 1:**
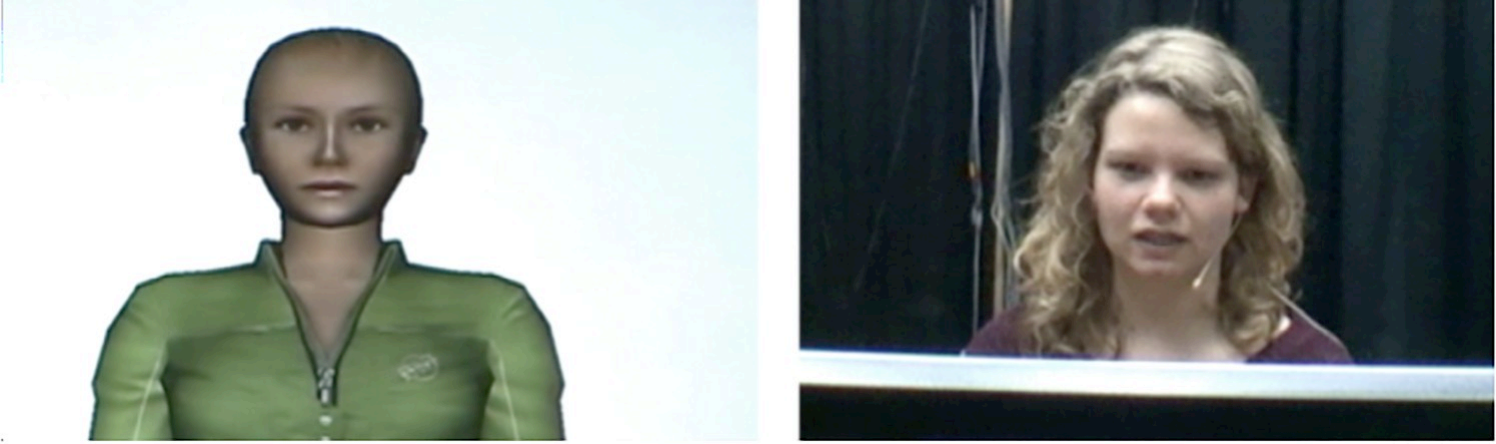
Virtual listener (left) interacting with human speaker (right) in the present experimental set-up (see also S2 Video).

The experiment took place in the Virtual Reality laboratory at the Max Planck Institute for Psycholinguistics in Nijmegen, The Netherlands. Participants were seated in front of a computer screen (HP Compaq LA2405WG) with speakers (Hercules XPS 2.010) wearing a lightweight, head-mounted microphone (DPA-d:fine-88). Three synchronized video cameras (Sony 3CCD Megapixel) were used to record the participants (1) frontally, and (2) laterally, as well as to record a separate computer screen showing precisely what the participant was seeing on their screen (i.e., the avatar), thus allowing us to temporally link participant and avatar behavior (see Fig 1). Audio was recorded using Adobe Audition CS6. Thus, each recording session resulted in three videos and one audio file, which were synchronized based on audible and visible markers (produced at the beginning of each block) and exported in Adobe Premier Pro CS6 (MP4, 25 fps).

In the control room next to the experiment room, a confederate (see Procedure) was seated in front of a keyboard (Apple MB110LL/B) and a computer screen (17"Acer AL732) showing the participant from a frontal view in real time. Audio from the participant’s head-mounted microphone was also directly transmitted to the control room and played via speakers (Alesis M1Active 520) in real time.

#### Avatar characteristics and behaviors

The experiment was programmed using WorldViz’s Vizard 5.5. Three different female avatars were created based on a stock avatar produced by WorldViz. The avatars’ speech was pre-recorded by three different female native speakers of Dutch and played at relevant points during the experiment. The avatars’ lip movements were programmed to match the amplitude of the pre-recorded speech files, creating the illusion of synchronization. The speech materials consisted of a general introduction (e.g., *Hoi*, *Ik ben Julia*, *leuk je te ontmoeten*!; ‘Hi, I’m Julia, nice to meet you!’ and *Ik heb een aantal vraagen aan jou*; ‘I have a couple of questions for you’) and a set of 18 open-ended questions (e.g., *Hoe was je weekend*, *wat heb je allemaal gedaan*?; ‘How was your weekend, what did you do?’). The avatar also gave a response to the participant’s answer (e.g., *Oh ja*, *wat interessant*!; ‘Oh, how interesting!’) before proceeding to the next open question (e.g., *Ik heb nog een vraag aan jou*; ‘I have another question for you’), or to close the interaction (*Hartelijk bedankt voor dit gesprek*, *ik vond het gezellig*!; ‘Thank you very much for this conversation, I enjoyed it!’) (see S2 Video for an example of a trial). Whenever an avatar was in the listener role, her feedback responses—the crucial experimental manipulation in the present study—were modeled on typical feedback behavior that occurs in natural conversation. These behaviors consisted of (1) a head nod accompanied by a short blink (208 ms from blink onset to blink offset) and (2) the same head nod but accompanied by a longer blink (607 ms from blink onset to blink offset). The durations were based on the average durations of short and long blinks in a corpus of Dutch face-to-face conversations [16] and the nods (duration: 499 ms from nod onset to nod offset) accompanied the avatar’s blinking behavior to mimic the typical natural environment of blinks occurring in feedback slots in conversation [16]. Since in our corpus data, the onsets of nods and blinks typically coincided (perhaps due to motoric synergies, i.e. the downward movement of the head potentially facilitating the downward movement of the upper eyelid [16]), the onsets of nods and blinks in the current study were programmed to coincide as well. Since the offsets of nods and blinks were relatively varied or uncoordinated in our corpus data, the offsets of nods and blinks in the current study were programmed in a way that impressionistically achieved the most natural look, resulting in long blinks lasting about 100 ms longer than the nods (see exact durations above). Crucially, the nods in the two conditions were identical in form and timing such that the only difference between the two conditions was the duration of the co-occurring blinks.

### Design

A within-subject design was used with listener feedback (none, nod with short blink, nod with long blink) as independent variable, mean answer length as the dependent variable, and Empathy Quotient as an individual difference measure. The experiment consisted of three blocks, one block per feedback condition and avatar. The set of 18 question stimuli were split up into three sets of 6 questions and each set was assigned to one of the three avatars, meaning each participant heard each question only once. The order of feedback conditions as well as the assignment of avatars (and thus the 6 questions that were paired with the respective avatars) to the listener feedback conditions was counterbalanced across participants. The order of items (question stimuli) within each block was randomized.

### Procedure

On arrival in the laboratory, participants were seated in front of the computer screen (see Fig 1) and were asked to meet and have a conversation with three different avatars and to respond to their questions. After a short personal introduction, the avatar asked questions and produced different types of visual feedback responses while participants answered (see Avatar characteristics and behavior section). The visual feedback responses were triggered secretly by a confederate, a Dutch native speaker who could see and hear the participant (via a video-camera link) but who could not see the avatar and the feedback behaviors being triggered. The confederate was blind to the experimental hypotheses and not informed about the manipulations.

The confederate was simply instructed to press a button whenever it felt appropriate to signal understanding while listening to the participants’ answers. Upon each answer completion by the participant, the avatar produced a response to the participant’s answer (e.g. ‘Oh, how interesting!’), which was also triggered secretly by the confederate. After having finished the conversation with the third avatar, the experiment was over and participants were asked to complete questionnaires (see above) before they were debriefed on the purpose of the experiment. Written informed consent was obtained before and after the experiment. The study was approved by the Social Sciences Faculty Ethics Committee, Radboud University Nijmegen. In line with PLOS ONE ethics regulations, written informed consent was obtained from all participants visible in still images or video footage included as part of this manuscript to publish the respective case details.

### Measures

#### Behavior coding

Answer length, the dependent variable in this study, was measured from the first vocalization (excluding in-breaths) to the last vocalization produced by each speaker in response to each question. Answers were always embedded between the offset of the avatar’s question and the onset of the avatar’s uptake, determined by when the confederate pressed the “Oh, how interesting!” button. Speech disfluencies such as “um” and “uh” were treated as being part of the answer. Answer lengths were annotated in ELAN 4.9.3 [25].

#### Questionnaires

Two questionnaires were used. Firstly, we used the Dutch version of the Empathy Quotient questionnaire first developed by Baron Cohen and colleagues, a widely used questionnaire measuring both cognitive aspects of empathy (i.e., understanding and/or predicting what someone else might think, feel, or do) and affective aspects of empathy (i.e., feeling an appropriate emotion triggered by seeing of another’s emotion; test-retest reliability: *r* = 0.97, *p* < .001 [22]). It consists of 60 statements (e.g., *I find it easy to put myself in somebody else's shoes* or *I can pick up quickly if someone says one thing but means another*) and participants indicate on a four-point scale to what extent they agree with each statement (strongly agree, slightly agree, slightly disagree, strongly disagree). Secondly, we used a questionnaire assessing any explicit awareness of the different feedback types, that is, whether participants had noticed nodding and/or blinking in the virtual listeners at all, and if so, if they had noticed any variation in these behaviors across conditions.

#### Statistical analysis

The answer length data set was trimmed for outliers deviating more than 2.5 standard deviations from the mean answer length (excluding 3% of the entire dataset; these were often cases where speakers started vocalizing, e.g. ‘uuhhm’, but then hesitated for a very long time thinking about what to answer). We used R [26] and *lme4* [27] to test in a linear mixed-effects model whether answer length differed depending on listener feedback. Our initial model was an intercept-only model estimating the mean answer length including intercepts for items (question stimuli) and participants as random effects. Using a likelihood ratio test (using the ‘anova’ function), this intercept model was compared to a model which differed only in that listener feedback (no feedback, nods with short blink, nods with long blink) was included as a fixed effect. To test whether any effect of listener feedback on answer length was modulated by the speakers’ empathy, we first entered listener feedback (no feedback, nod with short blink, nod with long blink) and speaker empathy (EQ score as a scaled and centered continuous variable) as fixed effects (without interaction term), and intercepts for items (question stimuli) and participants as random effects into the model. This model was then compared to a model that only differed in that listener feedback and speaker empathy were entered as fixed effects *with* interaction term, again using a likelihood ratio test (with the ‘anova’ function).

## Results

**Fig 2:**
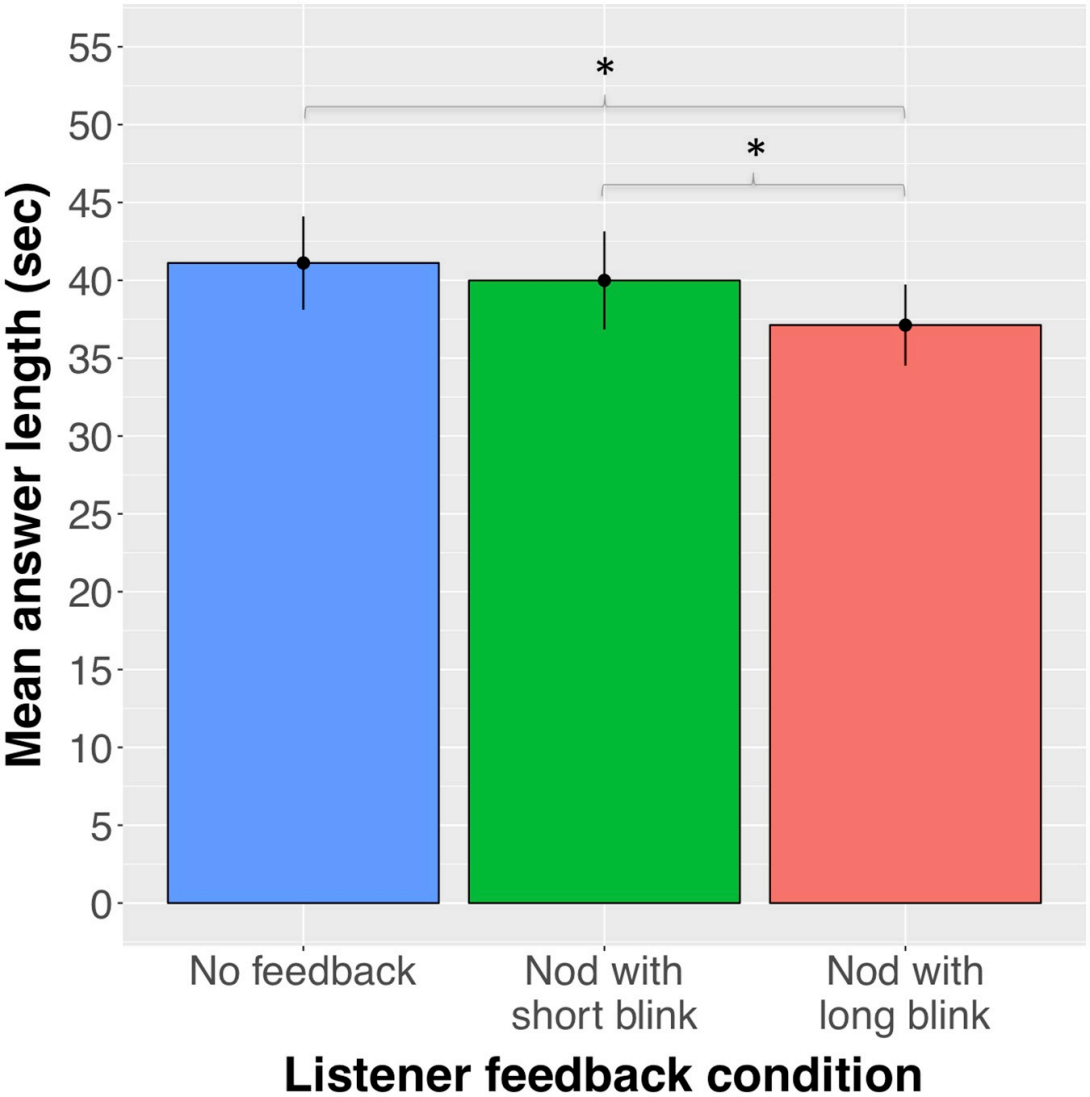
Mean answer length (sec) by listener feedback. Standard errors are represented in the figure by the error bars attached to each column.

Were speakers sensitive to listener blink duration in face-to-face communication? Our initial model was an intercept-only model estimating the mean answer length in seconds, including intercepts for items (question stimuli) and participants as random effects (*β* = 40.16, *SE* = 3.1, *t* = 12.95). Adding listener feedback as a fixed effect improved the model fit significantly (*χ*^*2*^(2) = 7.56, *p* = .022; AIC = 5187.2, BIC = 5213.8), revealing that, relative to talking to a listener providing feedback in the form of nods with short blinks (*β* = 40.82, *SE* = 1.68), speakers indeed produced shorter answers when talking to a listener providing feedback in the form of nods with long blinks (*β* = -2.86, *SE* = 1.42, *t* = -2.016, *p* = .044). Relative to talking to a listener providing no feedback (*β* = 41.7, *SE* = 3.2), speakers also produced shorter answers when talking to a listener providing feedback in the form of nods with long blinks (*β* = -3.74, *SE* = 1.41, *t* = -2.641 *p* = .008). Speakers’ answer length in the ‘nod with short blink’ condition and the ‘no feedback’ control condition were statistically indistinguishable (*β* = 0.87, *SE* = 1.41, *t* = 0.618, *p* = .537). These results show that speakers were not only sensitive to the absence of visual feedback, but also to subtle differences in blink duration (see Fig 2).

Note that predicting confederate’s feedback button press frequency (number of button presses per answer divided by the length of the same answer in minutes; *M* = 7.4; *SD* = 2.6) by feedback condition (nod with short vs. long blink), including random intercepts for participants and items, confirmed that button press frequency was consistent across conditions (*β* = 2.83, *SE* = 3.54, *t* = 0.801, *p* = .424).

Was speakers’ sensitivity to listener blink duration affected by their degree of empathy? We predicted answer length by entering listener feedback (no feedback, nod with short blink, nod with long blink) and speaker empathy (*M* = 39.56; *SD* = 10.66) as fixed effects (without interaction term), and intercepts for items (question stimuli) and participants as random effects into the model. This model was compared to a model that only differed in that listener feedback and speaker empathy was entered as fixed effects *with* interaction term. Including listener feedback and speaker empathy *with* interaction term did not improve the model fit significantly (*χ*^*2*^(2) = 3.808, *p* = .148; AIC = 5189.3, BIC = 5229.1). These results show that the effect of blink duration on answer length was unaffected by the speakers degree of empathy. The fact that not only high-empathizers but speakers in general were sensitive to differences in blink duration is in line with the hypothesis that listener blinking can indeed be perceived as a communicative signal.

Were speakers aware of the difference between short and long listener blinks? To address this question, participants were asked to complete a post-experiment questionnaire assessing whether they had noticed listener nodding or blinking. While all participants noticed the listeners’ nodding, only about half (46%) of the participants noticed the listeners’ blinking. Most importantly, none of the participants reported having noticed any variation in blink behavior across conditions, suggesting a covert effect of listener blink duration on speakers’ utterances.

## Discussion

Are speakers sensitive to listener blink duration in face-to-face communication? Our results reveal that subtle, millisecond differences in blink duration caused speakers to design answers to questions that differed substantially in length, namely by several seconds. Speakers produced shorter answers when talking to a listener providing feedback in the form of nods with long blinks instead of short blinks. Despite the striking effect on the speakers’ linguistic behavior, listeners’ blink behavior appeared to escape speakers’ explicit awareness.

These findings have theoretical implications. Although natural human language is multimodal and social-interactive in nature, traditional models of language processing have primarily focused on verbal language and on utterances produced outside of a social-interactive context. This study embraces the multimodal as well as the social-interactive nature of language and it may provide further motivation for a paradigm shift, an ‘interactive turn’ [28] that is already taking place in psycholinguistics [29–31], but also in the cognitive sciences more generally [32–35]. More specifically, our finding suggesting that long listener blinks may be functionally involved in managing mutual understanding highlights that speaking in interaction is not a unilateral process but a joint activity involving active contributions from both speaker *and* listener [1,2,4]. It further emphasizes that speaking not only involves self-monitoring [36] but also other-monitoring [37]. As Levelt [38] put it, “A speaker, while delivering his utterance, is continuously monitoring himself and his interlocutors, and this feeds back to what he is doing”. He further notes that “interlocutors send various signals to the speaker which tell him that (…) he should go on” and “much of this can be done by gaze or gesture”. The long listener blink as described in the present study appears to constitute such a gesture, a facial gesture that may be perceived as signaling “you’ve been sufficiently informative”. As such, the long listener blink is a type of backchannel [2]. More specifically, it is what Bavelas and colleagues [39] called a “facial backchannel”, a facial listener response providing “rapid feedback to the speaker without interrupting or taking up a turn”.

In the following, we discuss a number of open questions, limitations, and potential avenues for future research. So far, we have suggested that long listener blinks are perceived as a communicative signal of understanding. However, one may wonder whether the long listener blink is not merely a symptom (e.g., of low cognitive load) that is perceived by the speaker as communicative but not communicatively intended by the listener [40,41]. While this study does not address this issue conclusively, there are a number of reasons to believe that the long listener blink is indeed a communicatively intended signal of understanding. First, the longer duration of a long listener blink is an indicator of a voluntarily produced blink as opposed to a spontaneously produced blink, which is characterized by shorter durations [18]. Secondly, analyses of listener blinks in natural conversation [16] revealed that in contrast with short blinks, long blinks were more likely to be produced during the mutual gaze window [24] and they were produced in specific communicative contexts in which signaling understanding was especially relevant. Together, this suggests that long blinks may indeed be communicatively intended and specifically designed to signal ‘I understand, I’ve received sufficient information’ [42]. However, because our experiment was designed to test the consequences of perceiving listener signals on speaker behavior rather than the production of listener signals, we cannot be sure that long blinks were indeed used by listeners in this way. For example, one alternative possibility is that a nod with a long blink was simply interpreted as a signal to stop speaking, whether or not the answer had been understood. Thus, future experimental work is required to provide conclusive insights into the extent to which long listener blinks are communicatively intended, and what specifically they are meant to convey.

Another question that emerges from our findings is why speakers’ answer length did not differ when talking to a listener not providing any feedback (no feedback control condition) as compared to when talking to a listener providing feedback in the form of nods with short blinks. The nods with short blinks condition in our study was designed to be the ‘unmarked’ baseline condition, intended to signal that the listener currently has no special informational needs, i.e., that the speaker is neither under-telling nor over-telling [43–45]. The observation that absence of this sort of feedback does not affect speaker behavior seems at odds with previous research demonstrating the importance of listener feedback for speaking in face-to-face communication [4]. Note, however, that in that prior study, speakers interacted with a human listener who was distracted by a secondary cognitive task, resulting in deviant or absent listener responses at points where they were expected. In the current experiment, by contrast, speakers interacted with a virtual listener who did not provide any listener responses at all in the ‘no feedback’ condition. Presumably, if speakers did not get any feedback while answering the first question in the ‘no feedback’ condition, they may have assumed that this avatar is not “capable” of giving feedback at all, thus the speaker may not have expected any listener responses and produced his or her answers accordingly (as if talking to an answering machine). In fact, previous research has shown that expectations about the listener’s feedback can change the effect of listener feedback (or the omission thereof) [46]. It thus seems plausible that the participants’ expectations about the avatar’s behavioral repertoire may explain the lack of a difference in answer length between the short-blink and the no-feedback control condition. However, we should also bear in mind that, despite showing no difference in answer length, the quality of the answers may well have differed (e.g., answers in the no feedback condition may have contained more hesitations instead of semantic content). To explore this possibility, a subset of our speaker response data (n = 108) was coded for filled and unfilled pauses. These initial analyses did not suggest any differences in response quality, at least not with respect to filled and unfilled pauses. Since no substantial numerical differences were observed across conditions, the coding and analysis was not followed up for the remainder of the data due to the time-consuming nature of the analyses.

Since the current study has focused on the relatively global measure of answer length, future studies may zoom in on more immediate, local effects listener feedback may have on the content (e.g., level of detail, information density) as well as on speech production (e.g., hesitations, speech rate). Interestingly, at least impressionistically, there were no such effects on answer production. Also, a systematic analysis of hesitations for a subset of our data supported this impression, since it did not suggest any differences in the amount of hesitations produced across conditions (i.e., filled and unfilled pauses; see endnote), which may at least suggest that the shorter answers in the nod with long blink condition were not the result of speakers perceiving the avatar’s behavior as disruptive or interruptive.

On a related note, the main reason why speakers may have had varying expectations about the avatar’s feedback behavioral repertoire is that, in the current study, listener feedback was manipulated between avatars. The main reason to manipulate listener feedback between avatars was that we wanted to include a no feedback control condition. Since this is a novel paradigm, we needed a way to know whether speakers take into account listener feedback produced by an avatar at all (in case we had not found any differences in answer length between the nod with short versus nod with long blink condition). Thus, we manipulated listener feedback between avatars because we were concerned that interacting with an avatar providing human-like feedback in one trial but then suddenly no feedback in the next trial (control condition), may be too confusing for participants, disrupting the natural flow of the whole interaction with that avatar. Now that the current study has established that speakers are indeed sensitive to avatar listener feedback in general, and more specifically, to listener blink behavior, future studies may zoom in further on the moment-by-moment dynamics of the possibility of managing mutual understanding through blink feedback. Specifically, they may consider manipulating listener blink duration within individual virtual listeners, since in natural conversation, the same listener may sometimes display short blinks and sometimes long blinks, depending on situationally shifting informational needs. At this point, we can only speculate on the outcome of such a change in paradigm, but a likely possibility is that the contrast effect of perceiving both short and long blinks within one avatar may enhance the pattern of the current findings.

Taken together, our findings indicate that even visually subtle behavior such as listener blinking is anything but irrelevant in face-to-face communication. The different functions of listener blinking are, of course, not mutually exclusive. Like eye gaze in social contexts [47,48], it appears that eye blinking may serve self-oriented and other-oriented functions at the same time. In addition to physiological, perceptual and cognitive functions, listener blinks may serve a crucial communicative function in face-to-face communication. If this is true, cognitive and perceptual functions very likely preceded the communicative function, phylogenetically as well as ontogenetically. Blinking as a consequence of low cognitive load or attentional disengagement, and the need to control blinking to minimize audio-visual information loss during speech comprehension [49,50], may have been co-opted for communicative purposes through processes of ritualization [51,52], which would suggest a non-arbitrary, iconic relationship between form and meaning in communicative blinking [40,53]. Thus, the present findings may shed new light on the postulated “embodied” origin of the Understanding-Is-Seeing metaphor [54], and more generally, on the visual origins of mental-state signaling [55], a crucial ingredient for achieving intersubjectivity in everyday face-to-face communication. Moreover, they corroborate the notion that speaking in interaction is not a unilateral process but a joint activity involving active contributions from both speaker *and* listener [1,2,4].

## Supporting information

S1 Video Long listener blink. Example of a long listener blink as used in face-to-face conversation [16].
(MOV)

S2 Video Example of a trial (short blink). Example of a trial in the nod with short blink condition, including the avatar’s question, the avatar’s nods with short blinks during the participant’s answer, and the avatar’s response following answer completion.
(MOV)

S3 Video Example of a trial (long blink). Example of a trial in the nod with long blink condition, including the avatar’s question, the avatar’s nods with long blinks during the participant’s answer, and the avatar’s response following answer completion.
(MOV)

S1 Data Dataset underlying the findings. (CSV)

S1 Text Statistical model. (PDF)
