# Supplementary material for: Eye blinks are perceived as communicative signals in human face-to-face interaction
Source: PLoS One. 2018 Dec 12;13(12):e0208030. doi: 10.1371/journal.pone.0208030 (PMC6291193; doi:10.1371/journal.pone.0208030)
Supplement: S1 Text — (PDF) [file pone.0208030.s005.pdf]

Supplementary Information 5

R code for the model structure used to analyze the dataset underlying the findings (see Supplementary Information 4):

Estimating answer length by listener feedback condition

```
model1: Answerlength ~ 1 + (1|Participant) + (1|Item)

model2: Answerlength ~ Condition + (1|Participant) +
(1|Item)

anova(model1, model2)
```
